# Yellow Nail Syndrome Presenting With a Complete Clinical Triad: A Report of a Rare Case

**DOI:** 10.7759/cureus.100801

**Published:** 2026-01-05

**Authors:** Md Shamsuzzaman, Kazi Azad, Mubina Ahmed, Ekramul Mustafa

**Affiliations:** 1 Acute Internal Medicine, Mid and South Essex NHS Foundation Trust, Essex, GBR

**Keywords:** complete clinical triad, lymphedema, nail discoloration, pleural effusion, yellow nail syndrome

## Abstract

Yellow nail syndrome (YNS) is a rare disorder characterised by a triad of yellow nail discoloration, lymphoedema, and respiratory manifestations, including recurrent pleural effusions. A few hundred cases have been reported worldwide. Diagnosis is primarily clinical, as no definitive biomarkers exist, and the complete triad is seen in only a minority of patients. We report a 76-year-old male who presented with recurrent pleural effusions, non-pitting lower-limb oedema, and characteristic nail changes. Timely recognition of the classical triad allowed appropriate intervention, including indwelling pleural catheter placement and talc pleurodesis, leading to symptomatic improvement. This case highlights the importance of clinical vigilance in evaluating unexplained pleural effusions in older adults.

## Introduction

Yellow nail syndrome (YNS) is an uncommon multisystem disorder classically defined by the triad of yellow-discoloured, slow-growing nails, lymphoedema (most often of the lower limbs), and respiratory manifestations such as chronic cough, bronchiectasis, chronic rhinosinusitis, and recurrent pleural effusions. The syndrome was first described in 1964 and remains rare, with only a few hundred well-documented cases reported in the literature. The aetiology is incompletely understood, but lymphatic impairment or dysfunction is widely considered central to the pathophysiology and may explain the concurrence of nail changes, peripheral oedema, and effusions [1].

Clinical recognition is key because no specific biomarker exists, and the classical triad is often incomplete at presentation; large case series have reported that the complete triad is present in only 27-60% of patients [2]. Unlike common causes of pleural effusion, where systemic or radiological features often guide diagnosis, YNS may be overlooked unless nail changes and lymphoedema are actively identified. As a result, the condition may be misdiagnosed as onychomycosis, venous insufficiency, heart failure, or malignancy-related effusion. Reviews of published cases emphasise the variable expression of the triad and the predominance of respiratory involvement in many patients, often necessitating directed pleural management or lymphoedema care. Because pleural effusions in YNS are frequently recurrent, lymphocyte-predominant, and exudative, clinicians evaluating unexplained or recurrent effusions should include YNS in the differential diagnosis and examine the nails and limbs carefully [3].

## Case presentation

A 76-year-old man, a former smoker, was referred for evaluation of recurrent pleural effusions. His medical history included basal cell carcinoma, stable chronic kidney disease, hypertension, and hypercholesterolaemia. He reported progressive exertional dyspnoea and increasing bilateral lower limb swelling over several months, without fever, haemoptysis, weight loss, or recent travel.

**Figure 1:**
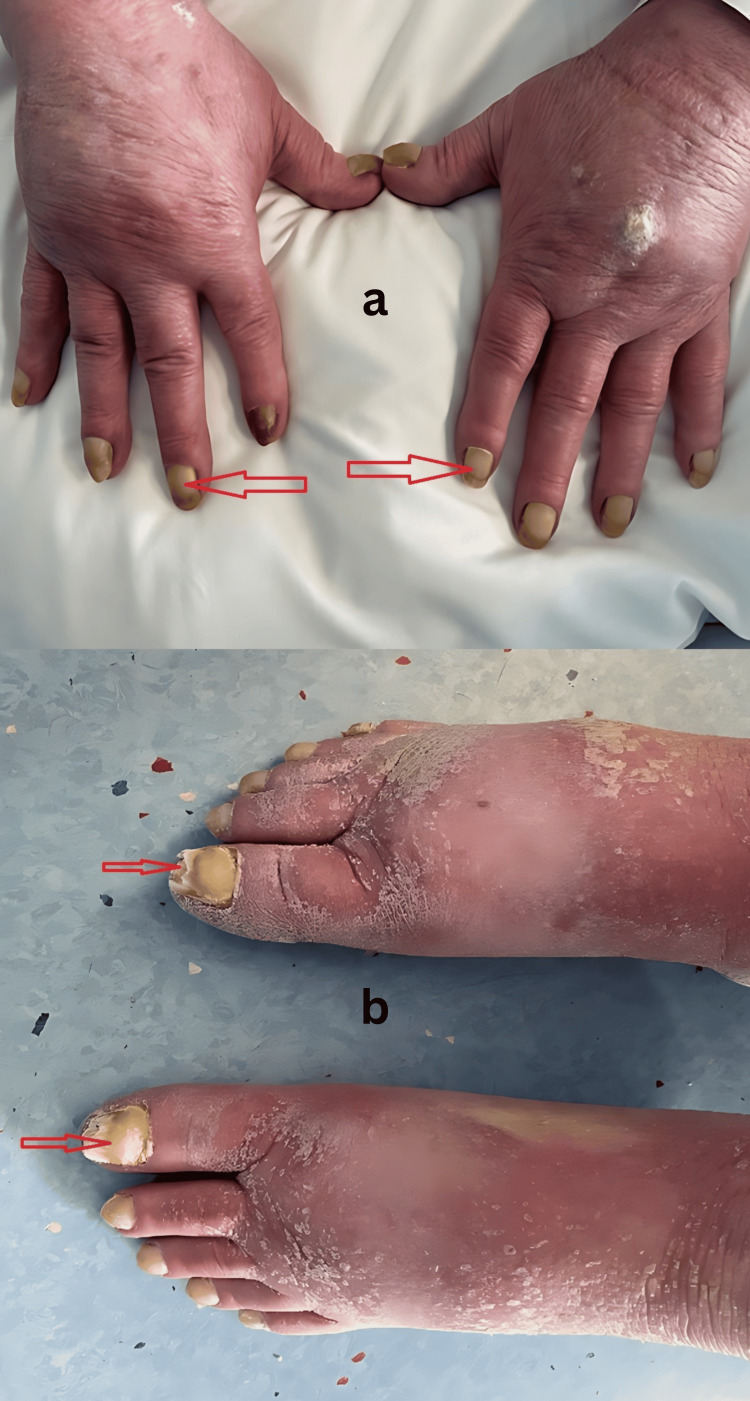
Dystrophic fingernails and toenails with yellow discoloration, thickened plates, and increased curvature.

**Figure 2:**
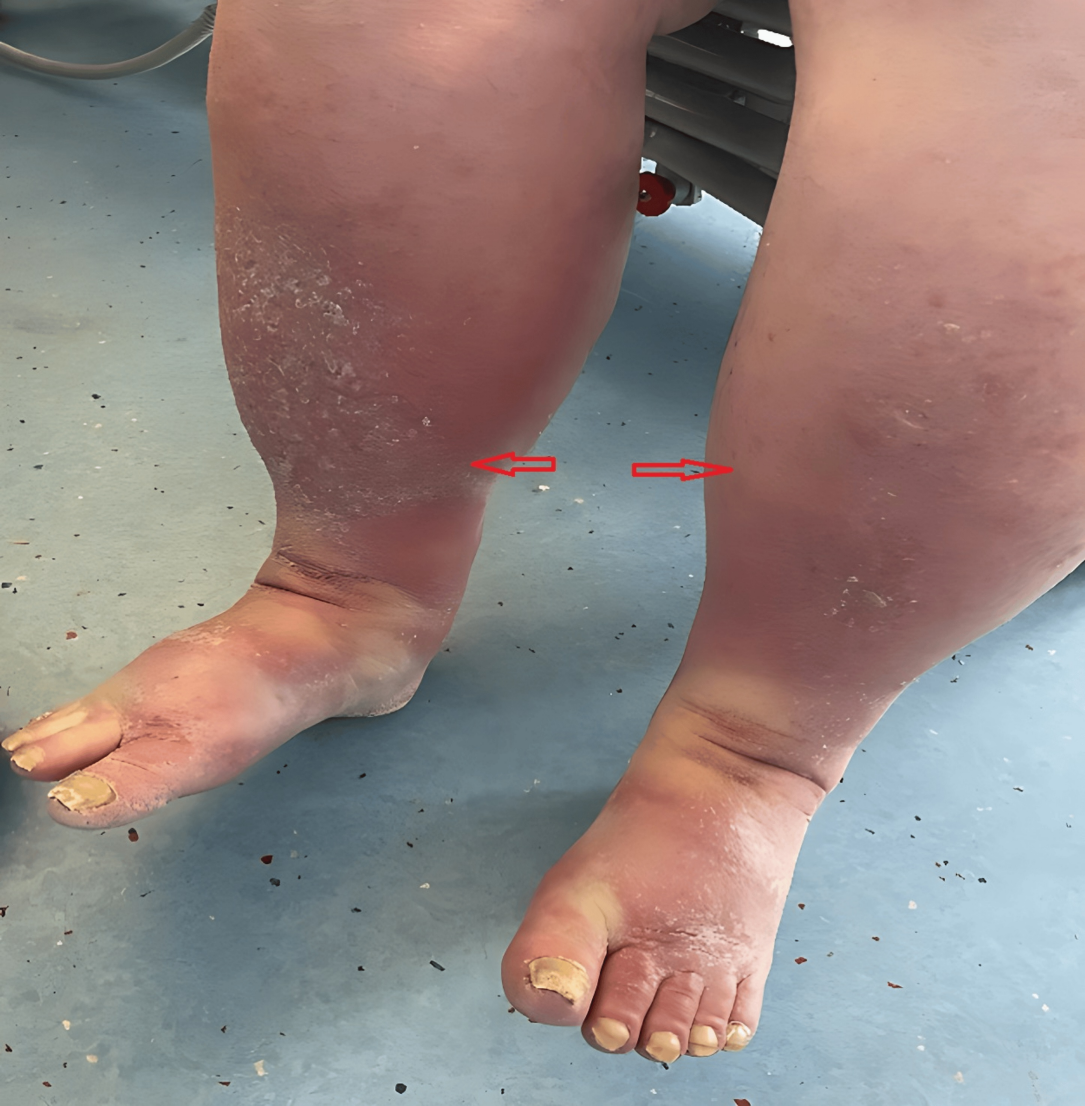
Severe chronic lymphoedema involving both legs.

On examination, he was afebrile and haemodynamically stable. Cardiorespiratory examination revealed dullness to percussion and reduced breath sounds at both lung bases. Nail inspection revealed diffuse yellow discoloration, thickening, and increased transverse and longitudinal curvature of both fingernails and toenails (Figure 1). There was no evidence of paronychia or periungual infection. However, non-pitting oedema was found on both lower limbs examination (Figure 2). 

**Figure 3:**
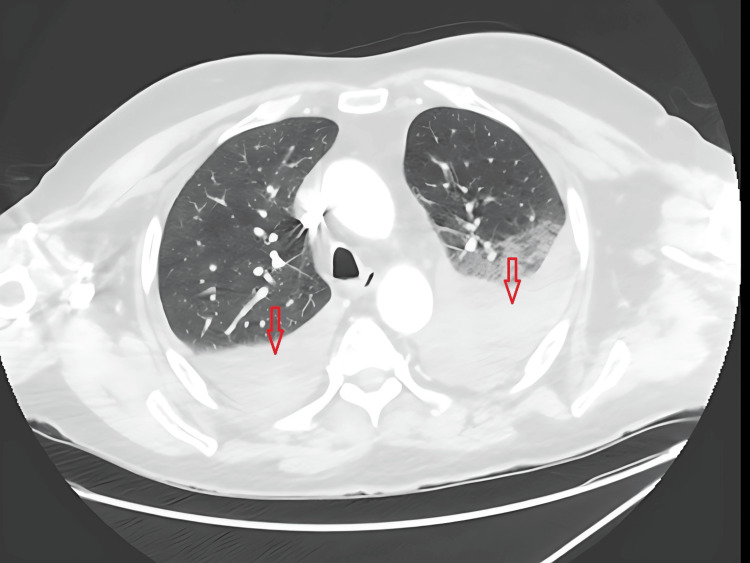
CT scan of chest showed bilateral pleural effusion

Chest radiography and computed tomography demonstrated bilateral pleural effusions without focal consolidation, bronchiectasis, or mass lesions (Figure 3). Diagnostic thoracentesis yielded a serous, exudative pleural effusion meeting Light’s criteria (fluid:serum protein ratio 0.67; fluid:serum LDH ratio 0.99). Pleural fluid cultures, mycobacterial testing, and cytology were repeatedly negative. Due to persistent reaccumulation, the patient underwent video-assisted thoracoscopic surgery. Pleural biopsy revealed reactive fibrous pleuritis with no evidence of granulomatous disease or malignancy.

Routine laboratory investigations, including full blood count, C-reactive protein, cardiac biomarkers, and transthoracic echocardiography, showed no evidence of cardiac failure. Liver and thyroid function tests were normal, and renal function remained at the patient’s established baseline. Autoimmune serology and an extended infective screen were unremarkable.

In the context of chronic nail dystrophy, bilateral non-pitting lymphoedema, and recurrent lymphocyte-predominant exudative pleural effusions with exclusion of infection and malignancy, a clinical diagnosis of YNS was made.

An indwelling pleural catheter was inserted for ambulatory management. Due to ongoing symptomatic effusion despite regular drainage, talc pleurodesis was performed via the indwelling pleural catheter tract. Following pleurodesis, the patient reported significant improvement in dyspnoea and reduced drainage requirements, with follow-up imaging demonstrating a marked reduction in free pleural fluid. Lymphoedema management, including compression hosiery and referral to physiotherapy, was initiated. On subsequent follow-up, sustained symptomatic improvement was reported. 

## Discussion

YNS is a rare multisystem disorder with incompletely understood pathophysiology. Impaired lymphatic drainage is considered central, although associations with autoimmune disorders, malignancies, and chronic infections have been reported. It predominantly affects older adults and is diagnosed clinically.

Nail abnormalities in YNS include yellow discoloration, thickening, and slowed growth, likely reflecting impaired nail matrix function. Respiratory manifestations can include recurrent pleural effusions, chronic sinusitis, and bronchiectasis. Lymphoedema, most commonly affecting the lower limbs, usually develops gradually. Rare cases have demonstrated mechanical lymphatic obstruction, such as mediastinal lipoma, confirming the role of lymphatic dysfunction in pathogenesis [4].

Management is largely supportive. Recurrent pleural effusions may be treated with repeated thoracentesis, indwelling pleural catheters, or chemical pleurodesis. Lymphoedema is managed with compression therapy and physiotherapy. Nail changes are often resistant to therapy, although spontaneous improvement has been described. Novel therapeutic options reported in recent case studies include oral minoxidil and terbinafine combined with topical minoxidil [5].

This case illustrates a rare presentation with the complete triad at diagnosis, highlighting the importance of early recognition to guide management and avoid unnecessary investigations.

## Conclusions

YNS is an uncommon but important diagnosis to consider in older adults presenting with unexplained or recurrent pleural effusions, particularly when nail dystrophy or peripheral lymphoedema is present. Because the classical triad may be incomplete or appear sequentially, careful physical examination, including inspection of the fingernails and toenails and assessment for lymphoedema, can be diagnostic and prevent unnecessary invasive investigations.

Management remains supportive and organ-targeted. Options for recurrent pleural effusion include therapeutic thoracentesis, indwelling pleural catheter placement for ambulatory symptom control, and chemical or surgical pleurodesis when appropriate. Early initiation of lymphoedema therapy may reduce morbidity. Nail changes show variable response to treatment, and evidence for pharmacological therapy remains limited.

We recommend consideration of YNS in lymphocyte-predominant recurrent exudative pleural effusions after exclusion of malignancy and infection, documentation of careful nail and limb examination, multidisciplinary management, and reporting of well-characterised cases with follow-up to improve understanding of disease course and treatment response.
